# Positive Emotional States in Dairy Cows: Reflections in Milk Quality and Udder Health

**DOI:** 10.3390/ani15223290

**Published:** 2025-11-13

**Authors:** Silvana Popescu, Daniela Elena Babiciu, Eva Andrea Lazar, Anamaria Blaga Petrean, Sorana Daina

**Affiliations:** 1 Faculty of Veterinary Medicine, University of Agricultural Sciences and Veterinary Medicine Cluj-Napoca, 400372 Cluj-Napoca, Romania; 2 Horse Welfare Association, 407207 Feiurdeni, Romania

**Keywords:** qualitative behaviour assessment, dairy cow behaviour, somatic cell count, affective states

## Abstract

Integrating positive welfare indicators into dairy science is gaining attention, yet the biological correlates of affective states in commercial herds remain poorly understood. This study explored associations between dairy cows’ emotional states quantified through the Positive Affect Index (PAI) derived from the Qualitative Behaviour (QBA) Assessment and milk biomarkers, yield, and udder health indicators across 37 commercial farms. Descriptive statistics, housing-adjusted linear regressions, and partial Spearman correlations were used to explore these relationships. Higher PAI values, indicating more positive herd-level emotional states, were significantly associated with lower SCC (22% reduction, *p* = 0.016) and lower odds of elevated DSCC (OR = 0.69, *p* = 0.002), reflecting improved udder health. Positive affect was also linked to a higher lactose concentration (*p* < 0.001) and an increased fat-to-protein ratio (FPR). A tendency for higher milk yield (+1.07 L per milking, *p* = 0.077) and slightly lower protein content was observed, consistent with a dilution effect. These associations remained robust after sensitivity analyses and were independent of housing type. The results demonstrate that milk composition and udder health biologically reflect positive emotional states, supporting the integration of behavioural assessments and milk biomarkers as a non-invasive framework for advancing welfare-oriented and precision dairy farming.

## Simple Summary

This study explored how the emotional state of dairy cows relates to milk quality and udder health under real farm conditions. Using a behaviour-based Positive Affect Index (PAI) derived from the Qualitative Behaviour Assessment (QBA), we evaluated 37 commercial dairy herds in Romania. The results showed that herds with more positive emotional states had lower somatic cell counts, indicating better udder health, and higher lactose levels, reflecting stable milk secretion. These herds also tended to produce slightly more milk, with only minor dilution effects on protein content. Overall, the findings suggest that the emotional well-being of cows is biologically reflected in their milk. Integrating QBA-derived indicators with routine milk biomarkers could provide a practical, non-invasive way to monitor and promote positive welfare within precision dairy management systems.

## 1. Introduction

Modern dairy-welfare frameworks emphasise not only the mitigation of negative states but also the promotion of positive welfare, creating opportunities for positive affect and behavioural expression at the herd level [1,2,3,4], with farmer perspectives highlighting feasibility and practical levers for implementation [5]. Within this paradigm, Qualitative Behaviour Assessment (QBA) provides a structured, integrative appraisal of the herd’s expressive quality, referring to the emotional tone and behavioural dynamics observed in the group. QBA has demonstrated reliability and validity across species [6,7,8,9,10] and is sensitive to environmental and management variation in dairy systems [11,12]. However, while QBA provides a robust behavioural measure of affect, its biological correlates under commercial farm conditions remain insufficiently explored.

In parallel, milk biomarkers, including the somatic cell count (SCC), differential SCC (DSCC), lactose, protein/casein, ketone bodies, and hygiene indicators (TPC), are routinely available as non-invasive signals of udder health, secretory function, and management hygiene [13,14]. These can be complemented by sensor-based physiological proxies, such as infrared thermography, that relate emotional arousal to production [15]. Despite progress on defining and measuring positive indicators [2,3], there is limited evidence on whether herd-level positive affect is reflected in milk-derived biomarkers under commercial conditions. Much of the existing literature has focused on negative states or experimental disease models (e.g., mastitis) [16]. Meanwhile, the measurable biological signals (such as hormonal, metabolic, or immunological markers) that reflect an animal’s positive emotional or affective state remain under-characterised. Moreover, structural features, notably housing systems (free-stall vs. tie-stall), can shape both behaviour and physiology [17,18,19,20], creating potential confounding that must be addressed to isolate affect–biomarker relationships. At the same time, precision-management approaches call for integrated, algorithm-ready dashboards that combine animal-based indicators with informative physiological metrics to guide proactive decisions [21,22,23,24].

Building on these developments, the present study aimed to (1) derive a Positive Affect Index (PAI) from positively valenced QBA descriptors using transparent standardisation and equal weighting, and (2) test whether herd-level positive affect is reflected in milk composition, udder health, and metabolic biomarkers at the farm level, while controlling for housing effects. We hypothesised that higher positive affect would be associated with lower inflammatory burden (SCC/DSCC) and greater lactose concentration, indicative of preserved epithelial/secretory integrity. In addition, we expected modest compositional shifts consistent with dilution at higher yield (slightly lower protein/casei), while no systematic associations were anticipated for energy-balance proxies (urea, ketone bodies) or hygiene (TPC). Methodologically, our goal was to provide a reproducible, non-invasive framework for integrating behavioural affect with routine milk data, consistent with the needs of precision livestock management and scalable across diverse farm contexts.

## 2. Materials and Methods

### 2.1. Study Population and Design

Between April 2023 and December 2024, data were collected from 37 commercial dairy herds in Transylvania, Romania, comprising a total of 3377 lactating cows. Herd sizes ranged from 13 to 340 animals, mainly Holstein-Friesian and Romanian Spotted breeds. The farms represented a range of production systems, including free-stall (26 farms) and tie-stall housing (11 farms), with variable use of pasture or outdoor loafing areas (OLA).

The farms were recruited with the assistance of breeders’ associations and field veterinarians based on the following criteria: commercial dairy herds with ≥10 lactating cows, the willingness of the farm owner to participate in the study, and the accessibility for repeated assessments and milk sampling.

### 2.2. Qualitative Behaviour Assessment (QBA)

The emotional expression of cows was assessed twice, at one-month intervals, according to the Welfare Quality^®^ Assessment Protocol for Cattle [25]. QBA captures the body language of animals and the integrative emotional tone of the herd. Depending on farm size and structure, between one and eight observation points were selected to cover different functional areas (feeding, resting, and milking) to capture behavioural diversity. At each point, cows were observed for periods ranging from 2.5 to 10 min, with a maximum total observation time of 20 min per farm [25].

Two trained observers conducted the QBAs. Before data collection, observers participated in a joint calibration session to harmonise descriptor interpretation. To ensure consistency and reduce variability in scoring, both observers visited for training, during which they independently evaluated the same cows. Inter-observer reliability for QBA scoring was then evaluated using the Interclass Correlation Coefficient (ICC, two-way random model, average measures), yielding an average ICC of 0.826, which indicates good agreement according to conventional benchmarks [26].

Following observation, the assessor scored 20 behavioural descriptors on 125 mm visual analogue scales (VAS) anchored between “minimum” (absent) and “maximum” (dominant). After each farm assessment, the descriptors (active, relaxed, fearful, agitated, calm, content, indifferent, frustrated, friendly, bored, playful, positively occupied, lively, inquisitive, irritable, uneasy, apathetic, happy, distressed, and sociable) were independently scored, with the possibility that multiple terms could receive high values (e.g., animals being both calm and content). For descriptors with negative valences, higher scores represented a more negative expressive state.

### 2.3. Milk Sampling and Analysis

Individual milk samples were collected from all lactating cows (*n* = 3377) during the morning milking on the same day as the QBAs. The two visit means were then averaged to obtain a single farm-level value per biomarker. This procedure ensured temporal alignment between milk sampling and behavioural assessment, reduced day-to-day variability, and preserved the statistical independence of farms.

All analyses were performed in a laboratory accredited according to the international standard ISO/IEC 17025 [27], using Foss analytical equipment (Foss, Hillerød, Denmark). Milk yield per milking was recorded during official milk recording and expressed as litres per cow per milking. Milk composition (fat, protein, casein, lactose) was expressed as g/100 g and determined by mid-infrared spectrometry (MilkoScan FT+). Urea was expressed in mg/dL. Somatic cell count (SCC) was measured by the fluoro-opto-electronic method (Fossomatic FC) and expressed as ×10^3^ cells/mL. At the same time, differential SCC (DSCC) was assessed by fluorescence flow cytometry (Fossomatic 7 DC) and expressed as %. Ketone bodies (β-hydroxybutyrate [BHB] and acetone) were quantified in mmol/L by mid-infrared spectrometry on the MilkoScan FT+ (CombiFoss platform), and total plate count (TPC) was determined in CFU/mL by flow cytometry (BactoScan FC+).

### 2.4. Data Processing and Variable Transformation

For statistical inference, the unit of analysis was the farm (*n* = 37). Measurements from individual cows were aggregated at the herd level, and the two farm visits were averaged into a single value per variable. This yielded one independent observation per farm, aligned the timing of behavioural (QBA) and milk measurements, and reduced short-term noise/pseudo-replication.

Variables with skewed distributions were transformed before analysis: SCC and TPC were analysed on the log_10_ scale, and DSCC on the logit scale (after conversion to proportions). Displayed values remain on the original scale unless stated otherwise.

### 2.5. Construction of the Positive Affect Index (PAI)

To capture herd-level emotional state, we constructed a Positive Affect Index (PAI) by z-standardising six positively valenced QBA descriptors (lively, playful, happy, content, inquisitive, and positively occupied) and averaging them with equal weights. The PAI was constructed exclusively based on six descriptors with clear positive valences because these terms most consistently represent genuine positive emotional states rather than neutral or context-dependent behavioural activation.

Each descriptor was first z-standardised across all farms to place scores on a common scale and prevent high-variance descriptors from dominating the index. The z-score for farm *i* and descriptor *d* was computed as follows:

$$z_{i,d}=\frac{x_{i,d}-\mu_{d}}{\sigma_{d}}$$

where $x_{i,d}$ is the average score of farm *i* for descriptor *d* (after averaging across the two visits), and $\mu_{d}$ and $\sigma_{d}$ are the across-farm mean and standard deviation for descriptor *d*, respectively.

The Positive Affect Index (PAI) for each farm was then calculated as the mean of the six z-scores:

$$PAI_{i}=\frac{1}{6}\sum_{d\in \left\{\mathrm{lively},\;\mathrm{playful},\;\mathrm{happy},\;\mathrm{content},\;\mathrm{curious},\;\mathrm{positively}\;\mathrm{engaged}\right\}}z_{i,d}.$$

This standardised index (PAI_sd) was used as a continuous predictor in subsequent analyses, such that each +1 SD represents a meaningful increase in herd-level positive affect. By definition, PAI = 0 indicates an average level of positive affect (sample mean), PAI = +1 indicates one standard deviation above the mean (clearly above-average affect), and PAI = −1 indicates below-average affect.

### 2.6. Statistical Analysis

The statistical analysis combined descriptive statistics, non-parametric tests, linear regression models, correlation analyses, and sensitivity diagnostics. Descriptive statistics (mean, standard deviation, median, range) were calculated for all variables.

Descriptive statistics were computed for all variables. Housing effects (free-stall vs. tie-stall) were initially compared using two-sided Mann–Whitney U tests with Benjamini–Hochberg false discovery rate (FDR) correction.

For inferential modelling, endpoints were pre-classified according to biological relevance. The primary endpoints were selected to capture the three central domains of interest: production performance (milk yield per milking), protein metabolism and technological quality (protein and casein), and udder health/inflammation (SCC and DSCC). The remaining variables were designated as secondary endpoints, as they provide additional, but less central, context: lactose, fat-to-protein ratio (FPR), fat, urea, ketone bodies (BHB, acetone), and total plate count (TPC). Accordingly, endpoints were defined as follows: primary—milk yield per milking, protein, casein, SCC (log_10_), and DSCC (logit); secondary—lactose, FPR, fat, urea, BHB, acetone, and TPC (log_10_ CFU/mL).

For each endpoint, the following linear model was fitted: outcome = β_0_ + β_1_·PAI_sd + β_2_·Housing + ε, where PAI_sd represents the z-standardised Positive Affect Index, and housing (free-stall vs. tie-stall) was included as a covariate. The intercept (β_0_) corresponds to a free-stall farm with mean PAI (PAI_sd = 0); β_1_ represents the expected change in the outcome per +1 SD increase in PAI; and β_2_ represents the difference between tie-stall and free-stall farms at mean PAI. For SCC, analyses were performed on the log_10_ scale, and effects are presented on the original scale as percentage change via (10^β1^ − 1) × 100. For DSCC, analyses used the logit of the proportion, and effects are presented as odds ratios, exp (β_1_).

For the primary endpoints, significance was assessed at α = 0.05. For the secondary endpoints, *p*-values were additionally corrected using the Benjamini–Hochberg FDR procedure. To provide a model-agnostic perspective, Spearman correlations were calculated between PAI_sd and all milk biomarkers; partial Spearman correlations, controlling for housing, were also performed.

Additional sensitivity analyses included the following: models for protein and casein, with milk yield added as a covariate (dilution check); robust regression (M-estimation) to evaluate the influence of outliers; and leave-one-out (LOO) refits to assess the stability of coefficients across farms. Model assumptions were checked using the Breusch–Pagan test for homoscedasticity, Cook’s distance (threshold 4/*n*) to identify influential farms, variance inflation factors (VIF < 2) to exclude collinearity, and natural cubic splines for PAI to test linearity.

All analyses were performed in R software (version 4.5.1, R Core Team, Vienna, Austria). Data handling used the tidyverse package; regression models were fitted using base stats; partial correlations were computed with ppcor.

## 3. Results

### 3.1. Housing-System Contrast

Descriptive comparisons between housing systems are summarised in Table 1.

Compared to tie-stall housing, free-stall farms generally exhibited a more favourable production and milk composition profile, characterised by higher yields and greater protein and casein contents. These differences decreased after FDR correction but remained directionally consistent.

No clear distinctions emerged for fat or lactose, while the fat-to-protein ratio (FPR) tended to be slightly higher in tie-stall herds, although not significantly.

The indicators of udder health (SCC and DSCC) showed a trend toward a lower inflammatory burden in free-stall herds. DSCC reached nominal significance (*p* = 0.036), remaining borderline after adjustment. The energy-balance indicators (urea, BHB, acetone) and hygiene (TPC) did not differ consistently between systems.

Notably, the Positive Affect Index (PAI) was significantly higher in free-stall herds (*p* = 0.007), suggesting a more positive affective climate where behavioural freedom was less constrained.

Overall, the housing contrast confirmed expected structural influences, justifying the inclusion of housing as a covariate in subsequent models.

### 3.2. Positive Affect and Primary Endpoints

Associations between Positive Affect Index (PAI) and primary outcomes, adjusted for housing system, are presented in Table 2.

Housing-adjusted models revealed significant associations between PAI and key udder health indicators (Table 2). A one-standard deviation increase in PAI corresponded to a 22% reduction in SCC (*p* = 0.016) and 31% lower odds of elevated DSCC (*p* = 0.002).

For milk production, a tendency toward higher yield (+1.07 L/milking; *p* = 0.077) was observed, accompanied by a small but significant decrease in protein content (−0.12 g/100 g; *p* = 0.013). Casein showed a similar, non-significant direction. Together, these changes suggest a dilution effect associated with increased milk production rather than a decline in protein synthesis.

The models explained between 14% and 28% of variance across farms (adjusted R^2^), typical for heterogeneous commercial datasets. Notably, associations for SCC and DSCC remained statistically robust on the appropriate model scales despite modest overall fit.

### 3.3. Positive Affect and Secondary Endpoints

As shown in Table 3, positive affect was strongly and positively associated with lactose concentration (β = +0.09; FDR < 0.001) and fat-to-protein ratio (FPR) (β = +0.06; FDR < 0.001). Both results remained significant after multiple testing correction. This pattern aligns with a stable secretory function and a dilution scenario at higher yields (FPR increase driven primarily by a protein dip rather than by fat elevation), consistent with the primary-endpoint results.

The association with fat was directionally positive but not significant, indicating no consistent shift in fat content attributable to affect alone once housing and multiple testing are considered. Urea, β-hydroxybutyrate, and acetone showed no significant associations after FDR control, suggesting that energy balance was not systematically linked to affective state at the herd level. Total plate count (analysed on the log_10_ scale) showed no significant association, consistent with management- and hygiene-driven variability rather than affect-related physiology.

The explained variance was highest for lactose (R^2^ = 0.313) and FPR (R^2^ = 0.256), indicating stable patterns for these milk components.

### 3.4. Model Diagnostics and Sensitivity

Model assumptions were satisfied for all primary endpoints (Table 4).

Residual variance was consistent with homoscedasticity across outcomes, and adding a natural-spline term for PAI did not improve fit, indicating that a linear specification for PAI was adequate. A small number of herds were flagged as potentially influential by Cook’s distance. However, refitting with robust regression (M-estimation) yielded PAI coefficients of the same sign and comparable magnitude, indicating that the main inferences are not driven by single farms. VIF for PAI was low across models, indicating negligible collinearity with housing or other terms. The diagnostics support the validity and robustness of the adjusted models: assumptions were met, influential cases did not alter conclusions, and the linear effect of PAI provided a stable and biologically coherent description of associations with the primary endpoints.

### 3.5. Partial Correlations Between Positive Affect and Milk Biomarkers (Housing-Adjusted)

Partial Spearman correlations, controlling for housing, confirmed the regression findings (Table 5).

Lactose and milk yield correlated positively with PAI, while DSCC (logit scale) and SCC (log_10_ scale) correlated negatively; all four associations remained significant after FDR correction. The fat-to-protein ratio exhibited a positive trend that did not survive FDR adjustment. No robust correlations emerged for TPC (log_10_), urea, BHB, acetone, fat, protein, or casein.

## 4. Discussion

This study provides empirical evidence that positive emotional states in dairy cows, quantified through a QBA-derived Positive Affect Index (PAI), are reflected in milk composition and udder health biomarkers under commercial farm conditions. As this study was observational and cross-sectional, the results should be interpreted as associations, rather than definitive evidence of causality, and experimental research is needed to establish causal pathways between affective state and udder health. Herds expressing a more positive affect showed a lower somatic cell burden, lower differential SCC (DSCC), and higher lactose content, indicating a biologically coherent pattern of improved mammary integrity and stable secretory function.

Higher positive affect aligned with more favourable udder health profiles, consistent with evidence that herd-level behavioural assessments can track aspects of health in commercial settings [28]. Furthermore, the QBA proved to be sensitive to environmental variation in housed cows [9] and has been piloted as a positive welfare measure in loose-housing systems [11]. Experimental mastitis models have also linked disease to more negative affective states on the QBA [16], supporting the biological plausibility of lower SCC and DSCC where the affective climate is more positive (Table 2).

A positive association between the affect index and lactose fits the interpretation of lactose as the osmotic driver of milk secretion and a practical marker of epithelial/secretory integrity, in line with multidimensional welfare frameworks that advocate combining behavioural indicators with physiological signals [13,14]. The documented sensitivity of QBA to context and daily routines [9,12,29] offers a mechanism whereby smoother cow flow and more positive human–animal interactions support more stable secretory function (Table 2 and Table 3), and aligns with sensor-based evidence that physiological arousal patterns relate to production [15].

The tendency toward a higher yield accompanied by small downward shifts in protein and casein is most parsimoniously explained by dilution at greater secretion, rather than deterioration of technological quality, an important nuance in the broader debate over production as a welfare proxy [30,31]. Links between disposition/temperament and performance reported elsewhere provide convergent support for this direction of effect [32,33].

The absence of systematic associations with energy-balance proxies (urea, ketone bodies) or with total plate count is consistent with reviews indicating that these indicators are dominated by nutrition, management, and equipment hygiene rather than by herd affect per se [14]. This pattern suggests that the detectable footprint of positive affect emerges more clearly along the integrity–inflammation axis than through metabolic or hygiene pathways in commercial herds (Table 3 and Table 5).

The finding that free-stall herds exhibited more favourable udder health and production profiles and higher affect, compared to those in restrictive housing systems, provides important context. This result echoes the established literature demonstrating the disadvantages of such confinement versus the welfare benefits of less constrained movement and behavioural expression [17,18,19,20], where farmers emphasise the feasibility and value of positive-welfare opportunities [5]. Beyond its role as a statistical covariate, housing conditions likely interact with affective states and milk outcomes. Tie-stall systems, by restricting movement and social contact, may constrain behavioural expression and reduce opportunities for positive affective experiences, thereby contributing indirectly to less favourable physiological profiles. In contrast, free-stall environments allow greater freedom of movement, exploration, and social interaction, factors that may promote a positive emotional tone and indirectly support udder health through improved welfare and lower stress-induced inflammation. Moreover, within free-stall systems, access to pasture has been associated with improved welfare metrics [34], reinforcing the value of management features that enable natural behaviour. These patterns support the importance of environmental design as a moderator of both affect and production-related outcomes [19,20]. Adjusting for housing was therefore essential to isolate associations between positive affect and milk biomarkers beyond structural differences (Table 1).

Our findings operationalise calls to move beyond the prevention of negative states toward the promotion of positive welfare using measurable positive indicators [1,2,3]. Integrating a behaviour-based index (PAI) aligns with precision livestock approaches that advocate for algorithm-ready dashboards for early triage and proactive decision-making [21,22,23,24]. This index is combined with a compact biomarker panel consisting of SCC, DSCC, lactose, and, context-dependently, FPR. The entire approach is coherent with field-oriented positive welfare pilots [11] and established farmer-reported priorities and constraints [5]. In parallel, the literature on sensory enrichment and stockmanship interventions reports concurrent improvements in productivity and welfare indicators [35,36,37]. Within free-stall herds, pasture access represents a complementary management lever consistent with this direction [34]. These findings suggest that a composite PAI SCC/DSCC–lactose signal could serve as a non-invasive, management-ready trigger within routine workflows. QBA demonstrates inter- and intra-observer reliability under standardised protocols [6] and day-to-day sensitivity [12]. The present design addressed these aspects through the temporal alignment of QBA with milk sampling and farm-level aggregation across two visits. Recent validations in applied settings [10] and evidence of environmental sensitivity [9,29] provide context for interpretation. From a statistical standpoint, checks for homoscedasticity, linear functional form, negligible collinearity, and robustness to outliers, together with leave-one-out refits, are consistent with best practice in observational studies on commercial herds.

Integrating PAI with SCC, DSCC, and lactose into a concise, easy-to-track dashboard can support the prioritisation of checks, resource allocation, and targeted interventions [21,22]. Future intervention studies that strengthen human–animal interaction, optimise cow flow and space, implement feasible enrichment, and evaluate pasture access where feasible should aim to test causality along the affect–inflammation–secretion axis [34,35,36,37]. In parallel, higher-frequency longitudinal monitoring is needed. The integration of behavioural sensors (e.g., video analytics, thermography) may enable the scaling of a PAI-SCC/DSCC–lactose composite within everyday practice [14,15,24], providing a behavioural dimension to algorithmic welfare assessment. These tools can support advisory services and farm managers in tracking welfare trends over time, evaluating the impact of environmental or management changes, and guiding resource allocation toward preventive welfare strategies.

## 5. Limitations

Although this study provides valuable insights, several limitations should be considered. First, its observational, cross-sectional design restricts causal inference, and the results should be interpreted as associations rather than definitive evidence of causality. Additionally, while housing type was controlled for, other factors such as herd size, breed composition, milking routine, and human–animal interaction quality were not fully captured, potentially influencing both behaviour and milk biomarkers. The use of the Qualitative Behaviour Assessment (QBA) involves some subjectivity, despite good inter-observer reliability (ICC = 0.826), which may introduce variability. Furthermore, the short duration of behavioural observations may not have captured all relevant behaviours, and the stability of affective states over time could not be assessed. Finally, while the current analysis focused on descriptors with positive valences to operationalise positive welfare, future work should also incorporate descriptors with negative valences (e.g., fearful, agitated, apathetic) to disentangle low positive affect from high negative affect. Such a bidirectional assessment would strengthen the validation of the QBA as a dual-axis welfare indicator and enhance its utility within precision-monitoring frameworks.

## 6. Conclusions

This study demonstrates that positive affective states in dairy cows, quantified through a QBA-derived Positive Affect Index (PAI), are biologically reflected in milk composition and udder health. However, as this was an observational, cross-sectional study, these results should be interpreted as associations rather than evidence of direct causation. Herds expressing higher positive affect showed lower somatic cell count and differential somatic cell count, higher lactose concentration, and compositional shifts consistent with increased milk secretion rather than impaired quality. These relationships remained robust after accounting for housing system and statistical sensitivity checks, indicating that the physiological footprint of positive affect emerges most clearly along the integrity–inflammation axis. No consistent links were found with metabolic or hygiene markers.

Practically, combining behaviour-based welfare indicators (PAI) with routine milk biomarkers (SCC, DSCC, lactose) offers a non-invasive, scalable tool for early detection and proactive welfare management in commercial herds. This integrative framework aligns with precision livestock principles, promoting data-driven approaches to positive welfare.

Future work should apply longitudinal and interventional studies to test causality and explore sensor-based automation of the PAI-SCC/DSCC–lactose composite, thereby establishing a predictive framework for integrating affective states into routine health and welfare monitoring on dairy farms.

## Figures and Tables

**Table 1:** Milk composition, udder health indicators, metabolic biomarkers, and Positive Affect Index (PAI) by housing system.

Variable	Free-Stall (=26)		Tied (*n* = 11)		*p* Value	*p*-Value adj.
	Mean ± SD	Median (Min–Max)	Mean ± SD	Median (Min–Max)		
Milk yield (L/milking)	12.04 ± 3.41	11.50 (7.00–25.00)	9.91 ± 1.22	10.00 (8.00–12.00)	0.012	0.060
Protein (g-100 g)	3.67 ± 0.27	3.63 (3.23–4.26)	3.34 ± 0.32	3.38 (2.90–3.79)	0.013	0.060
Casein (g/100 g)	2.90 ± 0.24	2.85 (2.50–3.44)	2.69 ± 0.24	2.66 (2.30–3.03)	0.025	0.084
Fat (g/100 g)	4.01 ± 0.45	3.93 (3.22–4.81)	3.81 ± 0.34	3.85 (3.06–4.22)	0.273	0.354
Lactose (g/100 g)	4.78 ± 0.12	4.79 (4.47–5.01)	4.71 ± 0.16	4.70 (4.44–4.94)	0.183	0.339
FPR	1.09 ± 0.09	1.10 (0.92–1.23)	1.14 ± 0.11	1.14 (1.00–1.34)	0.244	0.354
Urea (mg/dL)	23.76 ± 9.17	23.38 (7.87–37.03)	20.36 ± 11.94	18.54 (5.77–44.79)	0.27	0.354
BHB (mmoL/L)	0.029 ± 0.037	0.023 (0.00–0.19)	0.027 ± 0.033	0.019 (0.00–0.12)	0.628	0.628
Acetone (mmoL/L)	0.006 ± 0.018	0.00 (0.00–0.09)	0.008 ± 0.027	0.00 (0.00–0.09)	0.534	0.578
SCC (×10^3^ cells/mL)	431.1 ± 213.1	451.1(82.41–953.40)	597.3 ± 259.8	498.6 (205.40–941.63)	0.17	0.339
DSCC (%)	57.9 ± 13.7	59.9 (31.1–86.3)	67.7 ± 15.9	76.0 (30.0–82.3)	0.036	0.094
TPC (×10^3^ CFU/mL)	252.5 ± 199.3	93.0 (10–2321)	234.7 ± 204.6	114.0 (25–1050)	0.425	0.502
PAI	0.27 ± 0.50	0.30 (−0.62–1.06)	−0.63 ± 0.96	−0.65 (−1.88–1.09)	0.007	0.060

SD—standard deviation; median values were compared by Mann–Whitney U test; *p*-value ≤ 0.05 is significant.

**Table 2:** Housing-adjusted associations between PAI (per + 1 SD) and primary endpoints.

Outcome	β (95% CI)	*p*-Value	Effect (Back-Transformed)	Adj. R^2^
Milk yield (L/milking)	+1.07 (−0.11, +2.25)	0.077	-	0.14
Protein (g/100 g)	−0.12 (−0.21, −0.03)	0.013	-	0.28
Casein (g/100 g)	−0.06 (−0.13, +0.01)	0.110	-	0.14
SCC (log_10_)	−0.11 (−0.20, −0.02)	0.016	≈−22%	0.17
DSCC (logit)	−0.37 (−0.61, −0.13)	0.002	OR = 0.69	0.26

Linear models adjusted for housing system; SCC effect expressed as percent change; DSCC as odds ratio (OR).

**Table 3:** Housing-adjusted associations between PAI (per + 1 SD) and secondary endpoints (FDR-controlled).

Outcome	β (95% CI)	*p*-Value	FDR	Adj. R^2^
Fat (g/100 g)	+0.06 (−0.08, +0.21)	0.399	0.417	0.005
Lactose (g/100 g)	+0.09 (+0.05, +0.13)	<0.001	<0.001	0.313
FPR	+0.06 (+0.02, +0.09)	<0.001	<0.001	0.256
Urea (mg/dL)	−1.45 (−4.97, +2.06)	0.417	0.417	−0.017
BHB (mmol/L)	−0.01 (−0.03, +0.01)	0.231	0.277	0.001
Acetone (mmol/L)	−0.01 (−0.02, +0.00)	0.155	0.22	0.04
TPC (log10)	−0.18 (−0.43, +0.07)	0.165	0.22	0.036

Models adjusted for housing. FDR = false discovery rate; R^2^ values indicate variance explained by the model.

**Table 4:** Model diagnostics and sensitivity (primary endpoints).

Outcome	BP *p*-Value	Max Cook’s D	Influential Farms (*n*)	VIF (PAI)	Spline *p*-Value
Milk yield	0.26	0.54	1	1.39	0.11
Protein	0.76	0.10	0	1.39	0.62
Casein	0.80	0.10	0	1.39	0.67
SCC (log_10_)	0.38	0.17	2	1.39	0.75
DSCC (logit)	0.88	0.27	2	1.39	0.80

BP = Breusch–Pagan test; Cook’s D threshold = 4/*n*; VIF < 2 indicates low collinearity.

**Table 5:** Housing-adjusted partial Spearman correlations between PAI and milk biomarkers and production.

Outcome	rho	*p*-Value	*p*-Value adj.
Lactose (g/100 g)	0.556	<0.001	0.004
logit-DSCC	−0.526	<0.001	0.005
Milk yield (L)	0.455	0.0046	0.018
log10-SCC	−0.428	0.0083	0.025
FPR	0.317	0.056	0.134
log10-TPC	−0.187	0.267	0.534
Acetone (mmol/L)	−0.170	0.314	0.538
Fat (g/100 g)	0.146	0.387	0.581
Protein (g/100 g)	−0.097	0.569	0.758
Urea (mg/dL)	0.069	0.685	0.822
Casein (g/100 g)	−0.016	0.927	0.947
BHB (mmol/L)	−0.011	0.947	0.947

FDR = false discovery rate adjustment.

## Data Availability

The original contributions presented in this study are included in the article. Further inquiries can be directed to the corresponding authors.
